# Impact of Prolonged Exposure of Eleven Years to Hot Seawater on the Degradation of a Thermoset Composite

**DOI:** 10.3390/polym13132154

**Published:** 2021-06-30

**Authors:** Amir Hussain Idrisi, Abdel-Hamid I. Mourad, Muhammad M. Sherif

**Affiliations:** 1 Department of Mechanical Engineering, United Arab Emirates University, Al Ain 15551, United Arab Emirates; amir.hussain@uaeu.ac.ae; 2 National Water and Energy Center, United Arab Emirates University, Al Ain 15551, United Arab Emirates; 3 Mechanical Design Department, Faculty of Engineering, Helwan University, Cairo 11795, Egypt; 4 Department of Civil, Construction and Environmental Engineering, School of Engineering, University of Alabama at Birmingham, Alabama, AL 35294, USA; msherif@uab.edu

**Keywords:** E-glass/epoxy composite, seawater, durability, mechanical properties, microstructural analysis

## Abstract

This paper presents a long-term experimental investigation of E-glass/epoxy composites’ durability exposed to seawater at different temperatures. The thermoset composite samples were exposed to 23 °C, 45 °C and 65 °C seawater for a prolonged exposure time of 11 years. The mechanical performance as a function of exposure time was evaluated and a strength-based technique was used to assess the durability of the composites. The experimental results revealed that the tensile strength of E-glass/epoxy composite was reduced by 8.2%, 29.7%, and 54.4% after immersion in seawater for 11 years at 23 °C, 45 °C, and 65 °C, respectively. The prolonged immersion in seawater resulted in the plasticization and swelling in the composite. This accelerated the rate of debonding between the fibers and matrix. The failure analysis was conducted to investigate the failure mode of the samples. SEM micrographs illustrated a correlation between the fiber/matrix debonding, potholing, fiber pull-out, river line marks and matrix cracking with deterioration in the tensile characteristics of the thermoset composite.

## 1. Introduction

Glass fiber-reinforced polymer (GFRP) composites are commonly used in automobile, aerospace and marine industries because of their high strength to weight ratio, stiffness and durability. Furthermore, glass fibers are simple to manufacture, economical, less fragile and have high chemical resistance when compared with carbon fiber-reinforced polymers (CFRPs). However, GFRPs are prone to harsh environment conditions such as UV, seawater, alkaline and corrosive conditions [1,2,3,4,5,6]. At high temperatures, polymer-based composites trap water in voids and are often fused into hydroxyl radicals of the epoxy polymers [7,8]. The immersion of polymers in water leads to deterioration in the physical properties of composites [9,10,11,12,13]. This results in the inflation of the epoxy polymer, causing bond failure due to hydrolysis and plasticization [14,15,16]. As polymers are submerged in hot water, small cracks are initiated within the polymer matrix due to water absorption and osmotic edge rupture [17,18,19]. This increases the degradation of composites with immersion time. Therefore, it is essential to ensure the durability of FRP composites in the seawater environment. Nanofillers are an effective reinforcement that could close gaps and enhance the compositional intensity [20,21,22]. Several studies have been performed in different aging conditions to investigate the performance of epoxy-based composites. It has been observed that the flexural properties of FRP samples decreased due to seawater immersion [23,24].

Pavan et al. [25] illustrated the reduction in tensile strength of glass/epoxy from 200.83 MPa to 146.42 MPa, and 185.27 MPa for atmospheric and subzero temperatures aging for 3600 h, respectively. Yan et al. [26] noticed a similar reduction in the mechanical properties of flax-fabric/epoxy composites. The tensile strength of the specimens reduced by 31.1%, 28.3% and 22.6% under the immersion of alkaline (5% NaOH) solution, seawater and water, respectively. Silva et al. [27] studied the durability of GFRP laminates embedded in epoxy resin. The composite was exposed to saltwater at 30 °C, 50 °C and 65 °C for 5000 h (approximately 7 months). For the first 1500 h, the modulus behavior indicated that the prevailing damage mechanism was swelling. Then, between 1500 h and 2500 h, the elastic modulus behavior indicated that plasticization was the predominant damage mechanism, especially for samples aged at 30 °C. Mourad et al. [13] studied the combined effect of seawater and temperature on the durability of glass/epoxy and glass/polyurethane composites that were submerged for one year. The tensile strength of glass/epoxy and glass/polyurethane reduced by 1% and 19%, respectively, for specimens that were immersed for one year at 23 °C. The reduction in tensile strength increased to 6% and 31% for glass/epoxy and glass/polyurethane, respectively, as the solution temperature increased to 65 °C. The tensile strain and modulus of glass/epoxy were not affected significantly. However, the failure strain of glass/polyurethane reduced by 25% for the submerged specimens at 65 °C. Merah et al. [28] evaluated the effect of seawater and outdoor temperature on the tensile strength of GFRP composite for the immersion of 6 months and 12 months. The observed reduction was 9%, 28%, and 3.6% for tensile strength, tensile strain and tensile modulus, respectively, for specimens that were submerged for 6 months. While specimens exposed for 12 months experienced a decrease in strength, strain, and modulus was 12%, 32.7% and 5%, respectively. Chakraverty et al. [29] studied the effect of seawater conditioning for up to 12 months on the mechanical properties, such as the interlaminar shear strength (ILSS), tensile stress and failure strain, elastic modulus and glass transition temperature (Tg) of GFRP composites. The tensile stress was reduced to 79% after immersion for 6 months. However, a slow recovery was observed, and the tensile strength rebounded to 84% after immersion for 12 months. The ultimate strain decreased by 12% and 20% after 6 months and 12 months of seawater immersion, respectively. These reduction in values of ultimate stress and strain are due to the plasticization and swelling of the composite.

Chen Y. et al. [30] utilized the Arrhenius equation-based prediction model for predicting the durability of GFRP-reinforced concrete structures. The GFRP bars were immersed in a concrete solution at temperatures of 20 °C, 40 °C, and 60 °C. The short-term data were used for the accelerated aging tests. Mourad et al. [31] investigated the long-term effect of seawater and temperature on the durability of E-glass/epoxy and E-glass/polyurethane composite for the exposure of 7.5 years. It was observed that the tensile strengths of the E-glass/epoxy composite reduced by 6.3%, 24.1% and 48.9% after immersion in seawater at 23 °C, 45 °C, and 65 °C, respectively for the duration of 7.5 years. However, this reduction was 37.6%, 48.7% and 63.6%, respectively for E-glass/polyurethane. Table 1 summarizes the research related to E-glass/epoxy composite immersion at different temperature.

It is evident from the above literature that seawater exposure of FRP composite is limited to a few months. The main objective of this research is to conduct a long-term experimental investigation of the durability of FRP composites. E-glass/epoxy composite samples were exposed at different temperatures (23 °C, 45 °C and 65 °C) for the period of 132 months (11 years) in seawater. After exposure of the composites to the harsh environment, the tensile strength, tensile strain and young’s modulus of the specimens were measured. Furthermore, scanning electron microscopy (SEM) and differential scanning calorimetry (DSC) analysis were conducted to investigate the damage mechanism of the composites.

## 2. Experimental Procedures

**Figure 1:**
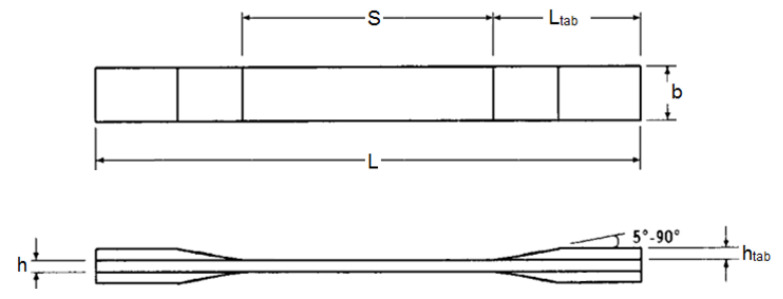
Line diagram of tensile test specimen.

In this research, the durability of unidirectional E-glass/epoxy composite reinforced with 52 vol% of glass fibers was investigated. The E-glass/epoxy was manufactured through a continuous lamination process and obtained from Gordon Composites, Inc. Samples with the thickness of 3 mm for the tensile test were fabricated as per ASTM Standard D-3039 [40]. The specimens had GFRP end-tabs bonded onto both ends to give uniform load distribution and to promote failure in the loading direction. The line diagram of the sample and its dimensions are given in Figure 1 and Table 2, respectively.

**Figure 2:**
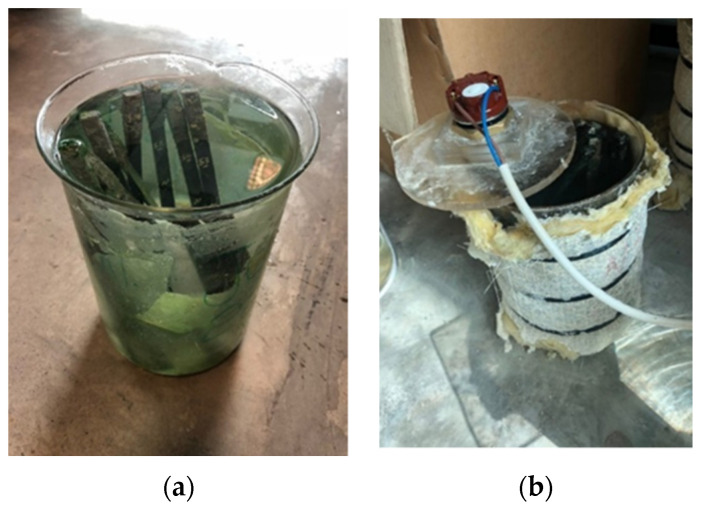
Representative images of the conditioning chambers (**a**) 23 °C, (**b**) 45 and 65 °C.

The shoulders of the samples were sealed by insulating tape to avoid any damage during the conditioning period. The samples were immersed in Gulf seawater at room temperature (23 °C), 45 °C, and 65 °C with an exposure time of 11 years. The tensile test results were recorded with an average of three test results of each group of specimens exposed to an environmental condition. The representative images of the conditioning chambers are shown in Figure 2.

## 3. Results and Discussion

### 3.1. Water Absorption

**Figure 3:**
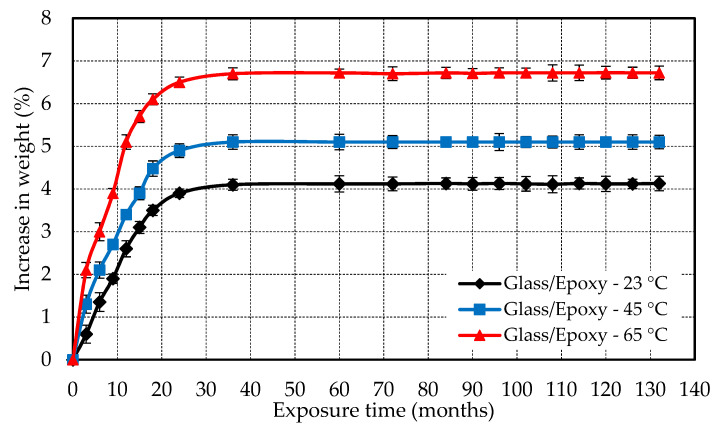
Variation in weight of E-glass/epoxy composite with conditioning duration and temperature.

The weight of specimens was measured to calculate the absorbed moisture before immersion and after the removal of the specimens from the water tank. Figure 3 illustrates an increase in the weight of the specimen was observed at 23 °C, 45 °C, and 65 °C for E-glass/epoxy specimens. The water absorption or increase in weight was 4.1%, 5.1% and 6.7% for samples immersed for 11 years at 23 °C, 45 °C, and 65 °C, respectively. It is worth noting that the water absorption increased steeply in the first 18 months of immersion. The rate of absorption decreased from 18 months to 36 months of immersion for E-glass/epoxy composites. After 36 months of immersion, the composite nearly saturated and the increase in water absorption was negligible for the remaining period of immersion. The increase in weight is due to the water absorption that occurs through diffusive and/or capillary processes. Diffusion is considered a key process under which moisture penetrates polymeric composites and is known to be a matrix-dominated phenomenon in which water is mainly diffused into the matrix [41,42]. The capillary process involves moisture being drawn into voids and microcracks at the fiber/matrix interfaces, in which the cracks provide a transport system for moisture to penetrate [43,44].

Moreover, it was observed that an increase in absorption rate occurs at as the exposure temperature is increased. This could be due to the degradation (i.e., cracks are initiated and crack growth rate increases) of the polymeric composite with the increase in temperature. Similar findings were reported in the literature, as an increase in temperatures has influence on accelerating the moisture absorption and degradation process in polymers [45].

### 3.2. Tensile Testing

**Figure 4:**
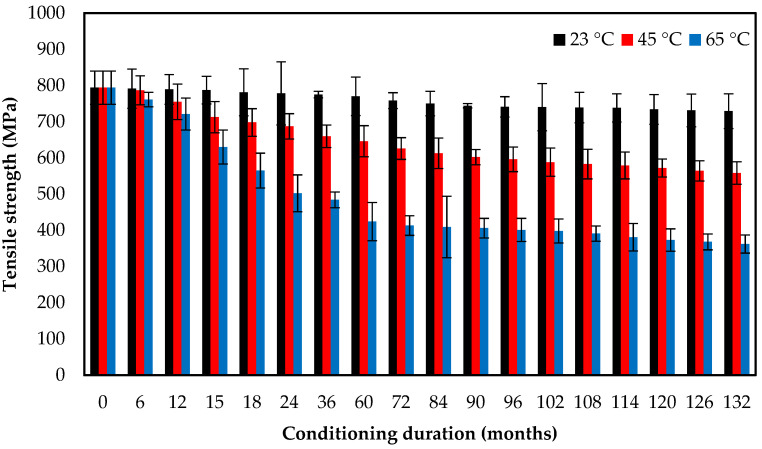
Tensile strength versus exposure time at different temperatures for E-glass/epoxy composite.

The tensile samples were manufactured according to the ASTM Standard D-3039 and tested at room temperature (300 K) using an MTS universal testing machine (MTS system corporation, Eden Prairie, MN, USA). The tests were conducted at a crosshead speed of 2 mm/min. The tensile strength of the samples after 11 years of exposure to seawater at different temperatures are summarized in Table 3 and Figure 4. The average of three test results for the same case is presented. The tensile strengths of the E-glass/epoxy composite after immersion in seawater at 23 °C, 45 °C, and 65 °C for the duration of 11 years was reduced from 794 MPa to 729 MPa, 558 MPa and 362 MPa, respectively. The reduction percentages are 8.2%, 29.7%, and 54.4%, respectively. These results show that exposure to seawater at elevated temperatures caused a higher reduction in the tensile strength than immersion in seawater at 23 °C [3,31,46,47,48,49,50,51]. The graph demonstrates that the tensile strength reduced by 46.6% after exposure for 5 years. However, a reduction in tensile strength of 14.6% was observed after exposure of 6 years and the immersion of specimens at 65 °C. The deteriorations in tensile strength could be ascribed to the combined effects of stress corrosion and diffusion of water molecules, resulting in the degradation of resin and weakening of the fiber/matrix interface. Furthermore, the chemical reaction between leaked metal ions (Na, K and Si) from E-glass fibers and Cl ions from seawater caused severe degradation to the tensile properties for samples submerged in seawater [52].

**Figure 5:**
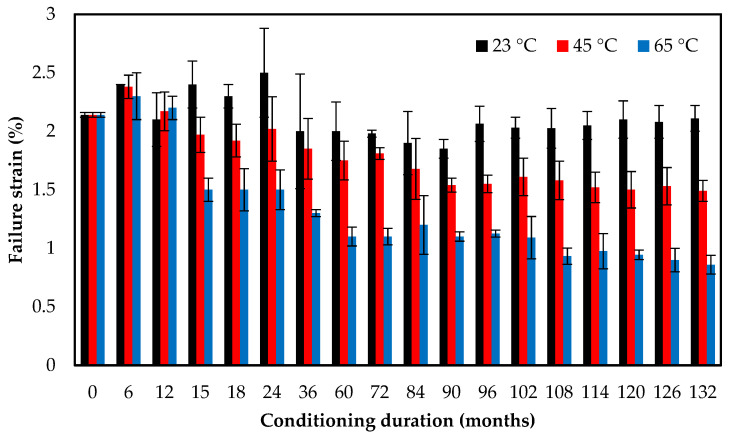
Tensile strain versus exposure time at different temperatures for E-glass/epoxy composite.

Figure 5 and Table 3 show the evolution of failure strain of the E-glass/epoxy composite after immersion in seawater at 23 °C, 45 °C, and 65 °C for the duration of 11 years. Figure 5 illustrates that after 2 years of exposure to seawater at 23 °C, the ultimate strain increased gradually from 2.14% to 2.50%. However, post 11 years of exposure, the specimens experienced a decrease in ultimate strain to 2.11%. Samples immersed at 45 °C had a similar behavior of an initial surge followed by a decline in failure strain, though at a faster rate. The ultimate strain for specimens submerged in seawater at 45 °C was 2.38% after 6 months of exposure which then decreased to 1.49% after 11 years of exposure. These observations illustrate that seawater uptake contributes to the plasticization of the epoxy matrix. This results in an increase in tensile strain and a decrease in tensile modulus. However, as the exposure duration increases, the matrix becomes more rigid and brittle, causing a decrease in failure strain and a slight increase in tensile modulus. This is more pronounced at 65 °C, as the tensile strain reduced to 0.86% after 11 years of immersion. Strait et al. [53] observed an increase in the impact energy of glass/epoxy due to initial plasticization at the early phase of seawater immersion. Additionally, Merah et al. [28] reported brittle fracture and a reduction in ultimate strain after prolonged exposure to seawater. In general, the prolonged seawater immersion causes plasticization and swelling in the composite material and affects the tolerance capacity of the composite material [29,54,55].

**Figure 6:**
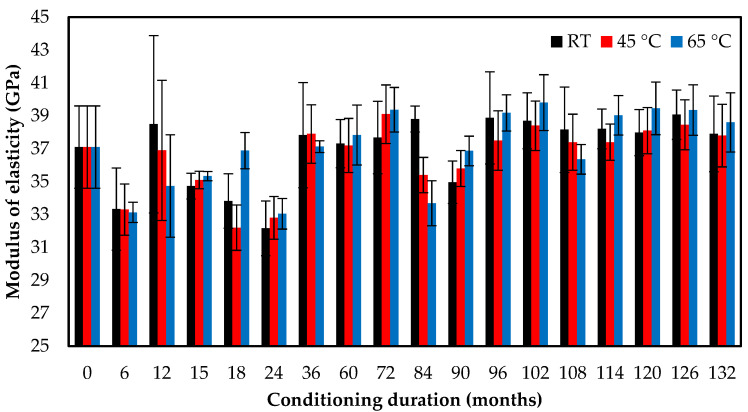
Effect of seawater immersion on the modulus of elasticity.

Figure 6 indicates that seawater immersion had a slight effect on the elastic modulus of the specimens exposed to various temperatures. The elastic modulus increased from 37.1 GPa to 37.9 GPa, 37.8 GPa, and 38.6 GPa for specimens immersed for 11 years at 23 °C, 45 °C, and 65 °C, respectively. The elastic modulus of the FRP specimens depended on the modulus of fibers. The immersion in the seawater environment had an insignificant effect on the durability of the fibers [56]. The data demonstrate that seawater immersion at the elevated temperature contributes to the epoxy matrix plasticization, which affects the failure strain and tensile modulus. Over a duration of 11 years of use, the matrix becomes stiff and fragile, allowing failure strain to decrease and tensile modulus to increase significantly.

**Figure 7:**
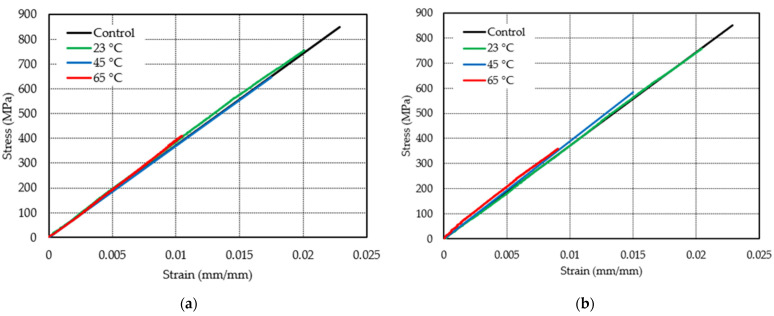
Stress–strain behavior of specimen immersed in seawater for (**a**) 5 years, (**b**) 11 years.

The effect of temperature in seawater conditioning was examined by the analogy of stress–strain behavior of the sample immersed for 5 years and 11 years at 23 °C, 45 °C, and 65 °C, as shown in Figure 6. The strength of specimens conditioned in seawater at 23 °C decreased by 3% after 5 years of exposure and 8.2% after 11 years of exposure, as shown in Figure 7a,b, respectively. The results show that the failure strain reduced by 6.5% and 1.4% after immersion at 23 °C for 5 years and 11 years, respectively. However, the modulus increased by 0.6% and 2.15%. At 45 °C, the tensile strength of the E-glass/epoxy composite declined by 18.6% and 29.7% for 5 years and 11 years of immersion, respectively, whereas the ultimate strain was decreased by 18.2% and 30.4%, respectively. The modulus of specimens exposed to seawater was almost similar to control specimens for 5 years of immersion but increased by 1.9% after 11 years of exposure. For immersion at 65 °C, the slopes of the graphs show an increase in tensile modulus by 2% from 37.1 MPa to 37.8 MPa of samples exposed to 5 years and 4% from 37.1 MPa to 38.6 MPa for samples exposed to 11 years. The failure strain of the specimen significantly decreased by 48.6% after 5 years of exposure and 59.8% after 11 years of conditioning at 65 °C, as shown in Figure 7. The reduction in tensile strength and failure strain could be due to the reaction between water and epoxy that causes a breakdown of the polymer’s molecular weight, leading to the fragile nature of the matrix. Water can also function as an anti-plasticizer, preventing polymer segments from moving and making the matrix more brittle [29]. Merah et al. [28] observed a decrease in brittle fracture and failure strain due to prolonged immersion in seawater.

### 3.3. Failure Analysis

**Figure 8:**
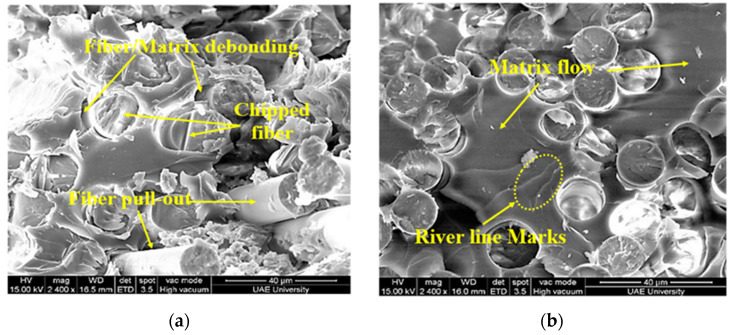
Fracture surfaces of the E-glass/epoxy composite conditioned for 5 years at (**a**) 23 °C (**b**) 65 °C.

**Figure 9:**
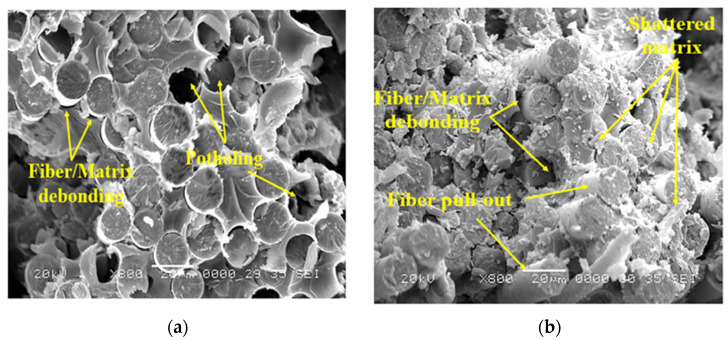
Fracture surfaces of the E-glass/epoxy composite conditioned for 11 years at (**a**) 23 °C (**b**) 65 °C.

The analysis of the fractured surface was carried out using the JEOL-JSM 7610F SEM (JOEL Ltd., Tokyo, Japan) and micrographs are presented in Figure 8 and Figure 9. The strong bonding between the fibers and the matrix was observed in E-glass/epoxy samples immersed for 5 years at 23 °C and 65 °C, as illustrated in Figure 8a,b. Slight deterioration in the matrix phase was noticed after the immersion of 5 years at room temperature, as shown in Figure 8a. At 65 °C, in Figure 8b, the bonding at the fiber–matrix interface further reduced and matrix flow and river line marks were observed; however, the fibers remained unaffected. This implies matrix breakdown occurred due to the hydrolysis reaction and accelerated at high-temperature immersion. Furthermore, it resulted in a non-uniform load distribution between fibers [13]. Few fibers were pulled out for specimens exposed for 5 years in seawater at 23 °C. After 11 years of exposure, significant fiber pullout and potholing was observed for composites immersed at temperatures of 23 °C and 65 °C, as shown in Figure 9a,b, which indicate the deterioration of the fiber/matrix interface. These images indicate a reduction in the bonding strength between the matrix and the fiber. Furthermore, as the immersion time increased, it resulted in a degradation in the tensile strength of the composite. It has been reported by Chen et al. [57] that the degradation of E-glass fibers involves mainly the etching of free hydroxyl ions (OH^-^) and leaching of water molecules. The free hydroxyl ions (OH^-^) break the Si-O-Si bond of the glass fibers, especially for etching in alkaline solution [57,58]. The damage at the fiber/matrix interface involves a complex mechanism as the fiber/matrix interface is a heterogeneous area [59,60,61,62,63]. Fracture at the fiber–matrix interface is usually caused by the debonding between fiber and resin which occurs mainly in two stages. The first stage includes the breakage of chemical bonding due to chemical corrosion between fiber and resin. The second stage occurs due to the poor interlocking between fibers and resin as the resin swells through water absorption [64].

Further degradation at the interface of the fiber–matrix is due to the large and prevalent gaps between fibers and matrices. The reaction between water and epoxy believed to cause a breakdown of the polymer’s molecular weight, leading to the fragile nature of the matrix. Water can also function as an anti-plasticizer, preventing polymer segments from moving and making the matrix more brittle [29]. Damage to glass fibers is not evident, but its smooth cross-sectional surface of the pulled-out fibers reveals brittle failure of the fiber. This could be due to the loss in ductility of the fiber, lack of support from the brittle matrix, or unhindered crack propagation into the fiber [13].

### 3.4. Differential Scanning Calorimetry (DSC) Test

**Figure 10:**
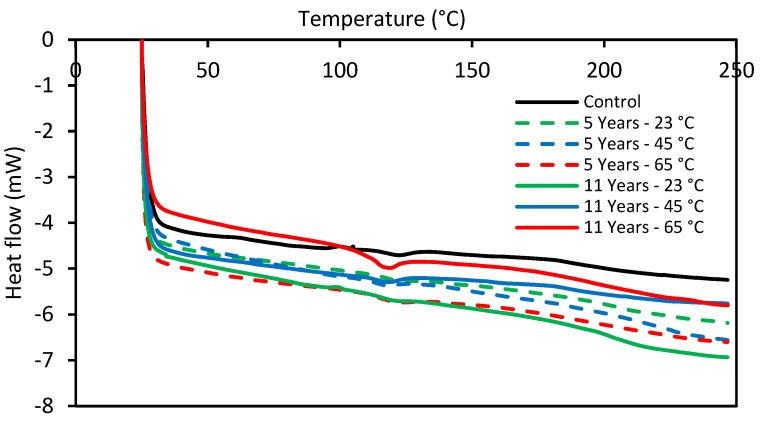
DSC curves for E-glass/epoxy control sample and conditioned samples.

The differential scanning calorimetry (DSC) for the E-glass/epoxy composite was conducted on samples immersed at different temperatures and exposure times. This test was carried out using a TA-Instruments DSC Q200 device (TA-Instruments, New Castle, DE, USA). The test was performed in an inert environment using nitrogen gas. The experiment was run between 25 °C and 250 °C with a heating rate of 10.0 °C/min. Three samples for each condition were considered to determine the glass transition temperature. Figure 10 represents the DSC curves for the control sample and specimens immersed at 23 °C, 45 °C, and 65 °C for 5 years and 11 years. The glass transition temperatures (Tg) of the samples immersed at 23 °C and 45 °C for 5 years and 11 years were found to be similar to the Tg of the control specimens (118.2 °C). The glass transition temperature of specimens immersed for 11 years at 23 °C and 45 °C were 117.72 °C and 117.11 °C, respectively. It indicates that the seawater immersion at 23 °C and 45 °C had a negligible effect on the Tg of the composite. However, a slight reduction in Tg was observed for specimens immersed in seawater at 65 °C. The Tg of samples reduced to 114.21 °C and 114.27 °C after immersion of 5 years and 11 years, respectively, at 65 °C. The shift in Tg could be due to the combined effect of high temperature and seawater aging. The behavior could be associated with the degree of cross-linking or the degree of polymerization after curing. The degradation in an epoxy composite is owing to the existence of hydrophilic groups which form weak hydrogen bonds by reacting with water molecules during immersion at 65 °C [65]. The results demonstrate that the moisture uptake acts as a plasticizer and decreases Tg. The reduction in Tg represents thermal degradation and loss in mechanical properties, which limits the service temperature of the polymer. The mechanisms of the long chain of the polymer could be isolated at high-temperature immersion and react with each other to alter the properties of the polymer [66].

### 3.5. Fourier Transform Infrared Spectroscopy (FTIR) Analysis

**Figure 11:**
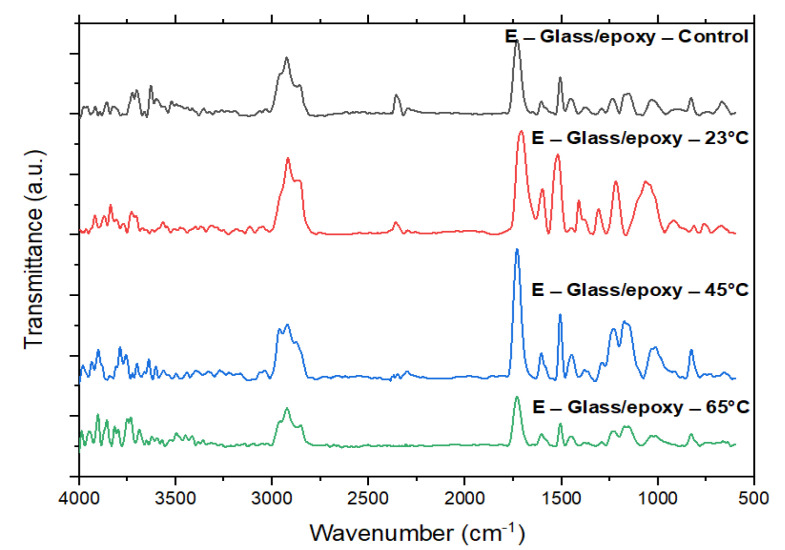
FTIR spectrum of E-glass/epoxy control sample and conditioned samples.

The Fourier transform infrared spectroscopy (FTIR) was performed on a Perkin Elmer Spectrum 100 FTIR spectrometer (PerkinElmer Life and Analytical Sciences, Shelton, CT, USA) at room temperature in the transmission mode. FTIR spectra were logged in between 600 cm^−1^ and 4000 cm^−1^ at a resolution of 2 cm^−1^ with 10 scans. Before testing the samples, background spectra were taken in the empty chamber to eliminate the influence of moisture and CO_2_ in air. Figure 11 presents a comparison of typical spectra for the control specimens of E-glass/epoxy and specimens immersed at 23 °C, 45 °C, and 65 °C for 11 years. The seawater exposure led to the presence of equivalent FTIR bands on the residue spectrum due to leaching of functional groups from the resin of composite.

The control E-glass/epoxy specimens had two peaks at 2296.3 cm^−1^ and 2353.2 cm^−1^. It indicates the presence of unreacted R-N=C=O groups [67,68]. The two peaks became weak after immersion of 11 years at 23 °C and 45 °C, but disappeared for specimens exposed to 65 °C. The vanishing of the two peaks at 2296.3 cm^−1^ and 2353.2 cm^−1^ after immersion for 11 years at 65 °C is due to the reaction between the water molecules and unreacted R-N=C=O groups, which can be presented as

R-N=C=O + H_2_O→R − NH_2_ + CO_2_↑ (1)

Therefore, the degradation at the fiber/matrix interface during immersion at 65 °C can be described as the release of CO_2_, shown in Equation (1). At this immersion temperature, the sustained release of CO_2_ is responsible for the increase in mass loss. This results in a reduction in the tensile strength of the composite [69,70,71,72].

## 4. Conclusions

In this research, E-glass/epoxy was conditioned at different temperatures (23 °C, 45 °C, and 65 °C) and exposure times (up to 11 years) to investigate the durability of the composite. The results show that seawater immersion slightly affects the durability of the composite at 23 °C; however, it reduced to more than half at 65 °C. The tensile strength of the E-glass/epoxy composites reduced by 8.2%, 29.7%, and 54.4% after immersion in seawater for 11 years at 23 °C, 45 °C, and 65 °C, respectively. The tensile strength reduced by 46.6% after exposure to seawater for 5 years, but the reduction was only 14.6% in the remaining 6 years of immersion at 65 °C. Similarly, the failure strain of the composites reduced with the increase in immersion temperature and exposure time. The degradation behavior was justified by the analysis of SEM micrographs, DSC curves and FTIR spectra. The deterioration was observed mainly owing to breakage of chemical bonding between fiber and resin due to chemical corrosion, and poor interlocking between fibers and resin due to resin swelling through water absorption.

## Figures and Tables

**Table 1:** Glass/epoxy composite immersed at different environment conditions.

Authors	Composite	Conditioning	Duration	Tests Performed
Silva et al. [27]	E-glass/epoxy	Saltwater at 30 °C, 45 °C and 55 °C	750–5000 hrs (Approx 7 months)	Water absorption, tensile properties
Chakraverty et al. [29]	E-glass/epoxy	Seawater at room temperature	2, 4, 6, 8, 10, and12 months (1 year)	Water absorption, ILSS properties, DSC, failure analysis
Hu et al. [32]	Glass/polydicycl-opentadiene and glass/epoxy	Saltwater and deionized water at 60 °C	1, 3, 6, and 12 months (1 year)	Water absorption, DMA, tensile strength, ILSS,
Guermazi et al. [33]	Glass/epoxy, carbon/epoxy and glass/carbon/epoxy	Tapwater at 24 ± 3, 70 and 90 °C	3 months	Water absorption, DMA, tensile and flexural test, abrasive wear test
Bobba et al. [34]	E-glass and S-glass fiber–epoxy	Tap water at 90 °C	600, 1200, and 1800 hrs (2.5 months)	Impact test
Feng et al. [35]	Glass/epoxy	H_2_SO_4_, NaOH and NaCl at 60 and 90 °C	7, 15, 30, and 90 days (3 months)	Water absorption, flexural properties, hardness, SEM analysis
Merah et al. [28]	Glass fiber-reinforced epoxy (GFRE)	Seawater at outdoor temperature	6 and 12 months	Tensile properties, failure analysis
Mourad et al. [13]	Glass/epoxy and glass/polyurethane	Seawater at 23 °C and 65 °C	3, 6, 9, and 12 months	Water absorption, tensile properties, DSC, failure analysis
Mourad et al. [31]	E-glass/epoxy and E-glass/polyurethane	Seawater at 23 °C and 65 °C	Up to 7.5 years	Tensile properties, failure analysis
EminDeniz et al. [36]	Glass/epoxy composite	Seawater at 20 °C	3, 6, 9, and 12 months	Impact test
Pavan et al. [25]	E-glass/epoxy laminates	Artificial seawater in sub-zero and ambient temperatures	3600 h(5 months)	Water absorption, tensile properties, and failure analysis
Wei et al. [37]	Basalt fiber-reinforced plastic (BFRP) and glass fiber-reinforced plastic (GFRP)	Artificial seawater at 25 °C	10, 20, 30, 60, and 90 days	Water absorption, tensile and flexural properties, and microstructural analysis
Antunes et al. [38]	Glass/epoxy filament wound cylinders	Seawater at 80 °C	7–28 days	Hoop stress, failure analysis
Ghabezi et al. [39]	Carbon/epoxy and glass/epoxy	Artificial seawater at room temperature and 60 °C	45 days	Tensile and 3-point bending

**Table 2:** Tensile specimen geometry.

Parameters	Dimensions
Specimen length, L	250 mm
Specimen width, b	15 mm
Specimen thickness, h	3 mm
Gauge length, S	150 mm
Tab length, L_tab_	50 mm
Tab thickness, h_tab_	4 mm
Tab bevel angle	90°

**Table 3:** Tensile properties of E-glass/epoxy after conditioning in seawater.

Conditioning Duration (Months)	23 °C			45 °C			65 °C		
	Tensile Strength (MPa)	Failure Strain (%)	Tensile Modulus (GPa)	Tensile Strength (MPa)	Failure Strain (%)	Tensile Modulus (GPa)	Tensile Strength (MPa)	Failure Strain (%)	Tensile Modulus (GPa)
0 (Control Sample)	794 ± 46	2.14 ± 0.02	37.1 ± 2.5	794 ± 46	2.14 ± 0.02	37.1 ± 2.5	794 ± 46	2.14 ± 0.02	37.1 ± 2.5
6	791 ± 54	2.4 ± 0.0	33.33 ± 2.5	786 ± 40	2.38 ± 0.1	33.3 ± 1.6	761 ± 20	2.3 ± 0.20	33.1 ± 0.6
12	789 ± 41	2.1 ± 0.23	38.49 ± 5.4	754 ± 49	2.17 ± 1.165	36.9 ± 4.3	721 ± 44	2.2 ± 0.1	34.7 ± 3.1
15	787 ± 38	2.4 ± 0.20	34.73 ± 0.8	712 ± 43	1.97 ± 0.15	35.1 ± 0.5	630 ± 47	1.5 ± 0.1	35.3 ± 0.3
18	781 ± 65	2.3 ± 0.1	33.82 ± 1.7	697 ± 38	1.92 ± 0.014	32.2 ± 1.4	565 ± 48	1.5 ± 0.18	36.9 ± 1.1
24	778 ± 87	2.5 ± 0.38	32.16 ± 1.7	687 ± 35	2.02 ± 0.275	32.8 ± 1.3	502 ± 51	1.5 ± 0.17	33.0 ± 0.9
36	775 ± 9	2 ± 0.49	37.83 ± 3.2	659 ± 31	1.85 ± 0.26	37.9 ± 1.8	484 ± 22	1.3 ± 0.03	37.1 ± 0.4
60	770 ± 53	2 ± 0.25	37.31 ± 1.5	646 ± 43	1.75 ± 0.165	37.2 ± 1.6	424 ± 53	1.1 ± 0.08	37.8 ± 1.8
72	758 ± 22	1.98 ± 0.03	37.68 ± 2.2	625 ± 30	1.81 ± 0.05	39.1 ± 1.8	413 ± 27	1.1 ± 0.07	39.7 ± 1.4
84	750 ± 34	1.9 ± 0.27	38.81 ± 0.8	612 ± 42	1.67 ± 0.26	35.4 ± 1.1	409 ± 85	1.2 ± 0.25	33.7 ± 1.4
90	744 ± 6	1.85 ± 0.08	34.96 ± 1.3	602 ± 21	1.54 ± 0.06	35.8 ± 1.1	406 ± 27	1.1 ± 0.04	36.9 ± 0.9
96	741 ± 28	2.06 ± 0.15	38.88 ± 2.8	596 ± 34	1.55 ± 0.075	37.5 ± 1.8	401 ± 32	1.13 ± 0.03	39.2 ± 1.1
102	740 ± 65	2.03 ± 0.09	38.7 ± 1.7	588 ± 39	1.61 ± 0.16	38.4 ± 1.5	398 ± 33	1.09 ± 0.18	39.8 ± 1.7
108	739 ± 42	2.03 ± 0.17	38.15 ± 2.6	583 ± 41	1.58 ± 0.165	37.4 ± 1.7	391 ± 21	0.93 ± 0.07	36.4 ± 0.9
114	738 ± 39	2.05 ± 0.12	38.21 ± 1.2	579 ± 37	1.52 ± 0.13	37.4 ± 1.1	381 ± 38	0.98 ± 0.15	39.0 ± 1.2
120	733 ± 41	2.1 ± 0.16	37.99 ± 1.4	572 ± 25	1.5 ± 0.156	38.1 ± 1.4	373 ± 31	0.95 ± 0.04	39.5 ± 1.6
126	731 ± 45	2.08 ± 0.14	38.4 ± 1.5	564 ± 28	1.53 ± 0.16	38.5 ± 1.6	369 ± 22	0.9 ± 0.1	39.4 ± 1.4
132	729 ± 48	2.11 ± 0.11	37.9 ± 2.3	558 ± 31	1.49 ± 0.09	37.8 ± 1.9	362 ± 25	0.86 ± 0.08	38.6 ± 1.8

## Data Availability

All data and models used during the study appear in the submitted article.
